# Bespoke microbiome therapy to manage plant diseases

**DOI:** 10.3389/fmicb.2013.00355

**Published:** 2013-12-03

**Authors:** Murali Gopal, Alka Gupta, George V. Thomas

**Affiliations:** Microbiology Section, Central Plantation Crops Research InstituteKudlu, Kasaragod, India

**Keywords:** microbiome, core-microbiome, plant, disease management, soil, rhizosphere, root, gut

## Abstract

Information gathered with advanced nucleotide sequencing technologies, small molecule detection systems and computational biology is revealing that a community of microbes and their genes, now termed “the microbiome,” located in gut and rhizosphere, is responsible for maintaining the health of human beings and plants, respectively. Within the complete microbiome a “core-microbiome” exists that plays the pivotal role in well being of humans and plants. Recent studies in medicine have shown that an artificial mixture of bacteria representing the core gut microbiome of healthy person when transferred into gut of diseased person results in re-establishment of normal microflora in the latter leading to alleviation from diseased condition. In agriculture, though not exactly in similar manner as in medicine, success in plant disease management has been achieved through transfer of microbiome by mixing disease suppressive soils with disease conducive soils. A study more similar to artificial gut microbiome transfer in medical field has been recently reported in agriculture, in which transfer of microbiome via soil solutions (filtered and unfiltered) has shown ability to alleviate drought stress in *Arabidopsis thaliana*. However, the exact practice of transferring artificially cultivated core-microbiome as in medicine has not thus far been attempted in plant disease management. Nonetheless, as the gut and rhizosphere microbiome are known to share many common traits, there exists a good scope for accomplishing similar studies in agriculture. Based upon the information drawn from all recent works in microbiome studies of gut and rhizosphere, we propose that tailor-made core-microbiome transfer therapy can be a success in agriculture too and it could become a viable strategy for management of plant diseases in future.

## Microbiome in relation to human and plant health

The power of next generation sequencing technology is transforming today's biology (Mardis, 2008; Schuster, 2008). Combined with bioinformatics (Lee et al., 2012), it is prising open the microbial “dark matter” and revealing the diversity and functions of microbiome at resolutions unknown hitherto (Forde and O'Toole, 2013; Rinke et al., 2013). It is shedding new light on the role played by the gut microbiome in governing the human health (Turnbaugh et al., 2007; Kinross et al., 2011; Cho and Blaser, 2012; Ottman et al., 2012; Norris et al., 2013), reviving the Metchnikoffian paradigm: colonizing the gut with beneficial microflora could lengthen the human life. The gut microbiota is not only limiting its influence on the human health by its functions in the intestine, it also is impacting the human brain and behavior (Heitz et al., 2011; Cryan and Dinan, 2012; Mulle et al., 2013) as well as social development evidenced by studies in mice (Desbonnet et al., 2013). Remarkably, similar train of evidences is being uncovered in plant world; root microbiome is observed to be tightly linked with the health of the plants (Friesen et al., 2011; Chaparro et al., 2012; Bulgarelli et al., 2013; Gaiero et al., 2013; Mendes et al., 2013). In insects, too, the same story is unfolding (Engel and Moran, 2013). The microbial diversity associated within these ecosystems is being referred to as the “second genome” that is easily 10 times more in scale than the host genome (Grice and Segre, 2012; Turner et al., 2013) and its impact on regulating human and plant health is becoming more apparent.

## Core microbiome

From among the multitude microbial communities inhabiting the gut and root, there appears to be a clutch of them which constitute the core microbiome (Tschöp et al., 2009). Core microbome contains organisms common across the microbiome hypothesized to play a key role in ecosystem function within a habitat (Lederberg and McCray, 2001). Core microbiome of human gut (Turnbaugh and Gordon, 2009; Turnbaugh et al., 2009; Huse et al., 2012; Petrof et al., 2013a,b) and plant (Bulgarelli et al., 2012; Lundberg et al., 2012; Peiffer and Ley, 2013) have been determined at Operational Taxonomic Unit (OTU) levels with small subunit ribosomal RNA genes or random sequencing of all genes. Any changes in the core-microbiome composition or function leads to debilitative or destructive diseases in humans as well as plants (Kinross et al., 2011).

## Disease suppressive soils and their microbiome

It is well known that farmers moved soil from one field to another to take advantage of its disease suppression abilities endowed by the soil microbial populations harbored in it (Weller et al., 2002). Soil microbial studies mainly based on cultivation dependent methods lead to the finding of several bacteria termed plant growth promoting rhizobacteria (PGPR) (Kloepper and Schroth, 1978), particularly the genus *Pseudomonas* spp., in imparting the disease suppressive ability to such soils (Schroth and Hancock, 1982; Haas and Defago, 2005; Mendes et al., 2013). Today, with advanced technologies, studies are generating evidence that it is not an individual or couple of microbes, rather it is “microbiome” (Forde and O'Toole, 2013; Rinke et al., 2013), the complete assemblage of microbial communities of a habitat and their functions, in rhizosphere that is determining plant health (Berendsen et al., 2012; Mendes et al., 2013; Rout and Southworth, 2013). In insects too, the same phenomenon is being observed (Hussa and Goodrich-Blair, 2013). Rhizosphere/core microbiome of *Arabidopsis* (Bulgarelli et al., 2012; Lundberg et al., 2012), desert shrubs *Zygophyllum dumosum* (Zygophyllaceae) and *Atriplex halimus* (Kaplan et al., 2013), and maize (Peiffer and Ley, 2013) have been deciphered and reported to be stable (Lozupone et al., 2012; Lundberg et al., 2012; Li et al., 2013), inheritable (Peiffer et al., 2013) and tightly linked to host tissues (Lee et al., 2013).

### Root microbiome transfer to manage plant disease

In plant disease management, a simple method of transferring complete microbiome by mixing disease suppressive soils with disease conducive one is practiced. Mendes and colleagues (2011) showed that when soils suppressive to *Rhizoctonia solani*, an important fungal pathogen, is mixed with disease conducive soils at 1:9 ratio (w/w), it successfully suppressed the infection in sugar beet. Metagenomic analysis of the soils using PhyloChip revealed consistent involvement of 17 bacterial communities belonging to Proteobacteria, Firmicutes, and Actinobacteria, considered as core-microbiome, in disease suppression. Other works too, similarly point to the involvement of core-microbiome in soils suppressive to potato common scab (Rosenzweig et al., 2012) and tobacco black root rot (Kyselkova et al., 2009). In all the above mentioned works, Pseudomonadaceae group of bacteria has been suggested as a key player in disease suppressiveness within the core microbiome.

### Gut microbiome transfer to manage human health

As with rhizosphere microbiome of plants in agriculture, in medical studies too, the gut microbiome has been found to control the health of the human beings (Turnbaugh et al., 2007; Cho and Blaser, 2012) with a core mainly involved (Tschöp et al., 2009; Turnbaugh et al., 2009; Huse et al., 2012). “Stool transplant” therapy (de Vos, 2013) is one of the several medical practices that is adopted wherein stool taken from healthy person is transferred to diseased person resulting in suppression of many important gastro-intestinal diseases. The principle here is to re-establish normal gut bacteria in the gut of diseased person and bring about positive changes in their health. However, the “stool transplant” therapy is not widely followed since the method is not acceptable to many patients, besides the apprehension that it can transfer pathogenic microbes too. Two recent studies (Petrof et al., 2013a; Ridaura et al., 2013) have found a way to overcome “stool transplant therapy” by using “stool substitute” in which a culturable consortium representing core microbiome is transferred and found to transmit the phenotype expression aimed for. Petrof and colleagues' (2013a) work was first of its sort successfully demonstrating that patients suffering from *Clostridium difficile* infection, a debilitative disease of intestine, can be cured when administered with stool substitute mixture comprising a multi-species community of bacteria (RePOOpulate sample) of a healthy individual exhibiting resistance to the disease. Post-treatment metagenomic analysis of the cured patients revealed that the OTU reads from their guts were similar to that of the RePOOPulate sample until six months after its administration even though the microbiota profiles were different. This work was quickly followed by Ridaura et al. (2013) in which they transplanted intact uncultured or cultured human fecal microbiota from each member of a discordant twin pair (one lean and other obese) into separate groups of recipient germ-free mice and found that the obese twin's fecal microbiota significantly increased the body biomass and adiposity in the germ free mice. It will not, therefore be, contrary to consider that the “stool substitute” transfer consisting of the core-microbiome is an extension of the “stool therapy” and is able to reproduce the expected microbial ecology with desired results. Such successful scientific endeavors are spurring development of new disease management paradigm termed MET: Microbial Ecosystem Therapy (Petrof et al., 2013b).

## Root can follow the gut

In agriculture, a system of manipulating the root environment by artificially inoculating plant and soil beneficial microbes has been followed for long time for improving crop yields. The PGPRs and other plant beneficial microbes (nitrogen fixing and phosphate solubilizing bacteria, *Trichoderma* spp., arbuscular mycorrhizae fungi etc.) isolated from rhizospheres were mass multiplied and artificially inoculated, either singly or in combination of twos, for disease management in plants (Berg, 2009; Lugtenberg and Kamilova, 2009; Chaparro et al., 2012; Qiu et al., 2013). Though this approach has been widely adopted, its success in field conditions have been limited (Bakker et al., 2012). With unequivocal reports coming out indicating that it is not a single taxon, but a consortium of microorganism that is responsible for bringing about diseases suppression in plants (Mendes et al., 2011; Rosenzweig et al., 2012; Trivedi et al., 2012; Klein et al., 2013), the stage is now set for the root to follow gut by adopting the strategy of using “stool substitute” for disease management. Transferring disease suppressive soils has been the only alternate method for transferring the complete rhizosphere/core microbiome in plant protection strategy. Coming closer to “stool substitute” therapy, transfer of microbiome via soil solutions (filtered and unfiltered) has shown ability to alleviate drought stress in *Arabidopsis thaliana*. Pyrosequencing analysis of soils revealed a core microbiome (*Burkholderia, Phormidium, Bacillus, Aminobacter, Acidiphilum* among others) involved in alleviating the abiotic stress (Zolla et al., 2013). However, the exact transfer of artificially cultivated core-microbiome as performed by Petrof et al. (2013a) and Ridaura et al. (2013) with gut environment is yet to be attempted in root environment. The possibilities of achieving success is high as there exists a striking similarity between the gut and root microbiota (Berendsen et al., 2012; Ramírez-Puebla et al., 2013). Also, the fact that soil type plays significant role in the selection and election of microbiome of rhizosphere and root compartment (Berg and Smalla, 2009; Bulgarelli et al., 2012; Lundberg et al., 2012), chances of success for the “rhizosphere substitute” is significantly augmented.

## Root microbiome culturing

In an important meeting convened on topic “culturing a plant-microbiome community” in Rhodes, Greece in 2012, a long-term future research strategy became apparent in which it was suggested that after an initial culture-independent survey of the plant microbiota, the corresponding community members are isolated in collections of pure cultures (Lebeis et al., 2012). Today, by converging information deduced on microbial diversity and functions using next-generation sequencing technologies and multi-species transcriptome analysis (Schenk et al., 2012), molecules/volatile involved in plant-microbe interaction using mass-spectral investigations (Watrous et al., 2012; Badri et al., 2013) combined with power of bioinformatics (Lee et al., 2012), it has become very much possible to culture the appropriate core-microbiome and apply it successfully (Ridaura et al., 2013). To assemble a robust core microbiome of an ecosystem not limited to OTU records alone, Shade and Handelsman (2012) and Lozupone and colleagues' (2012) suggested collecting the data on (i) OTU membership/α diversity, (ii) OTU composition/β diversity, (iii) OTU persistence across time and space and (iv) communication/metabolic networking among the OTUs. Determination of models, particularly of root environment, in which the plants favor the recruitment of antibiotic-producing (and -resistant) bacteria by stimulating interference competition through production of abundant resources, can help improve establishment of the artificially introduced microbiomes(Scheuring and Yu, 2013).

## Bespoke microbiome therapy for plant disease management

Artificial core-microbiome transfers can decrease the noise intrinsic to any complex communities and are step in right direction in disease management, both for plants and humans, built upon the principles of binary plant/human-microbe interaction in an ecological perspective.

The similarities between the gut and rhizosphere microbiota is striking in many aspects which can encourage emulating experiments carried out in gut with root environment and vice-versa. Based on the increasingly available body of evidences discussed in this article, we propose the model of transfer of bespoke core-microbiome, rather than individual species, as a viable strategy for management of plant diseases in future.

### Conflict of interest statement

The authors declare that the research was conducted in the absence of any commercial or financial relationships that could be construed as a potential conflict of interest.
